# Supplementary material for: Clinical Presentation and Prognostic Features in Patients with Immunotherapy-Induced Vitiligo-like Depigmentation: A Monocentric Prospective Observational Study
Source: Cancers (Basel). 2022 Sep 21;14(19):4576. doi: 10.3390/cancers14194576 (PMC9558529; doi:10.3390/cancers14194576)
Supplement: Supplementary file 1 [file cancers-14-04576-s001.zip › Suppl Figures S1+S2.pdf]

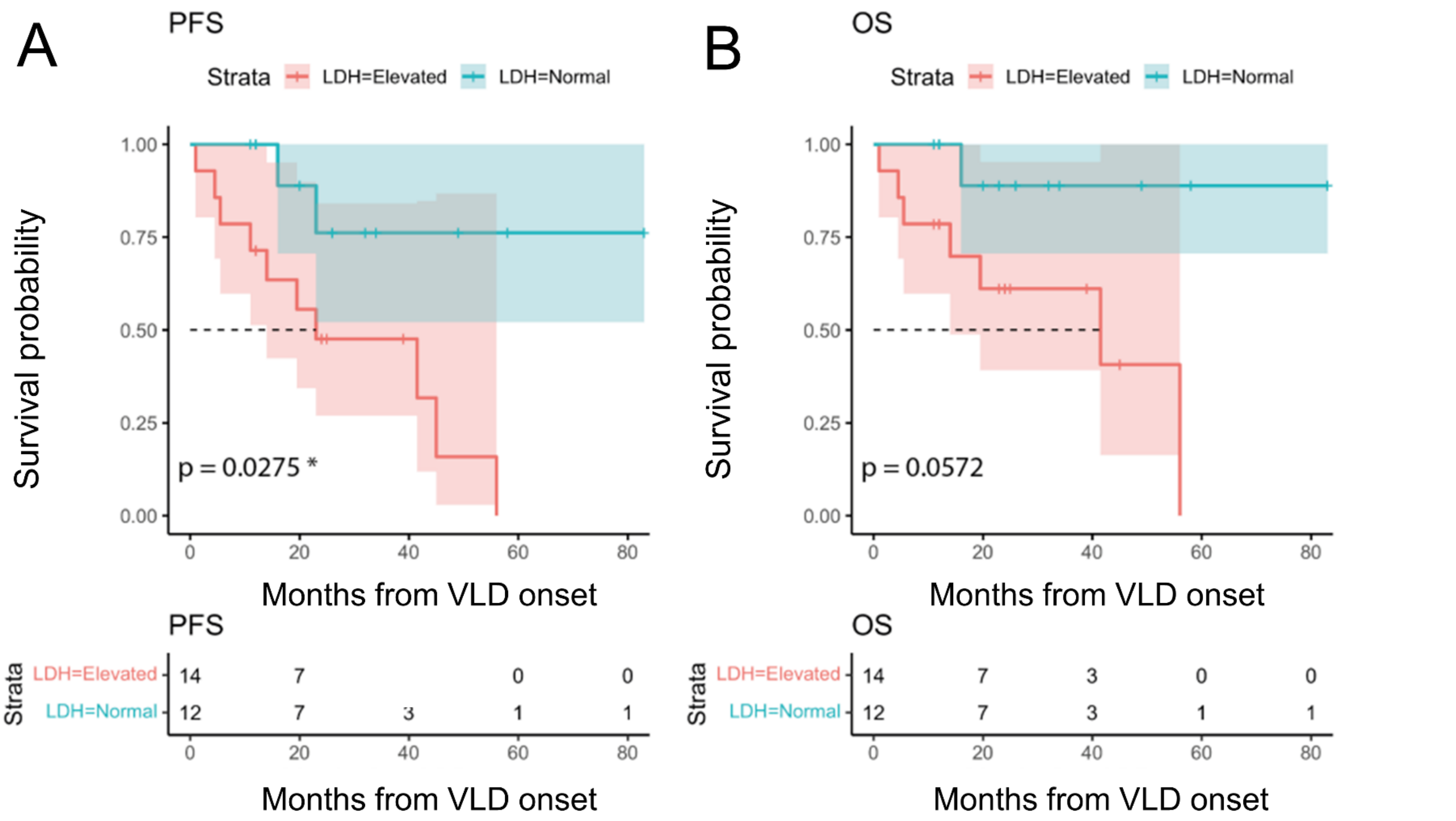

Supplementary Figure S1: Survival analysis dependent on LDH levels
(A) Progression-free-survival in normal LDH showing a significantly better prognostic value than with elevated LDH (p = 0.03). (B) Overall-survival in normal LDH showing a borderline-significant better prognostic value than with elevated LDH (p = 0.06).

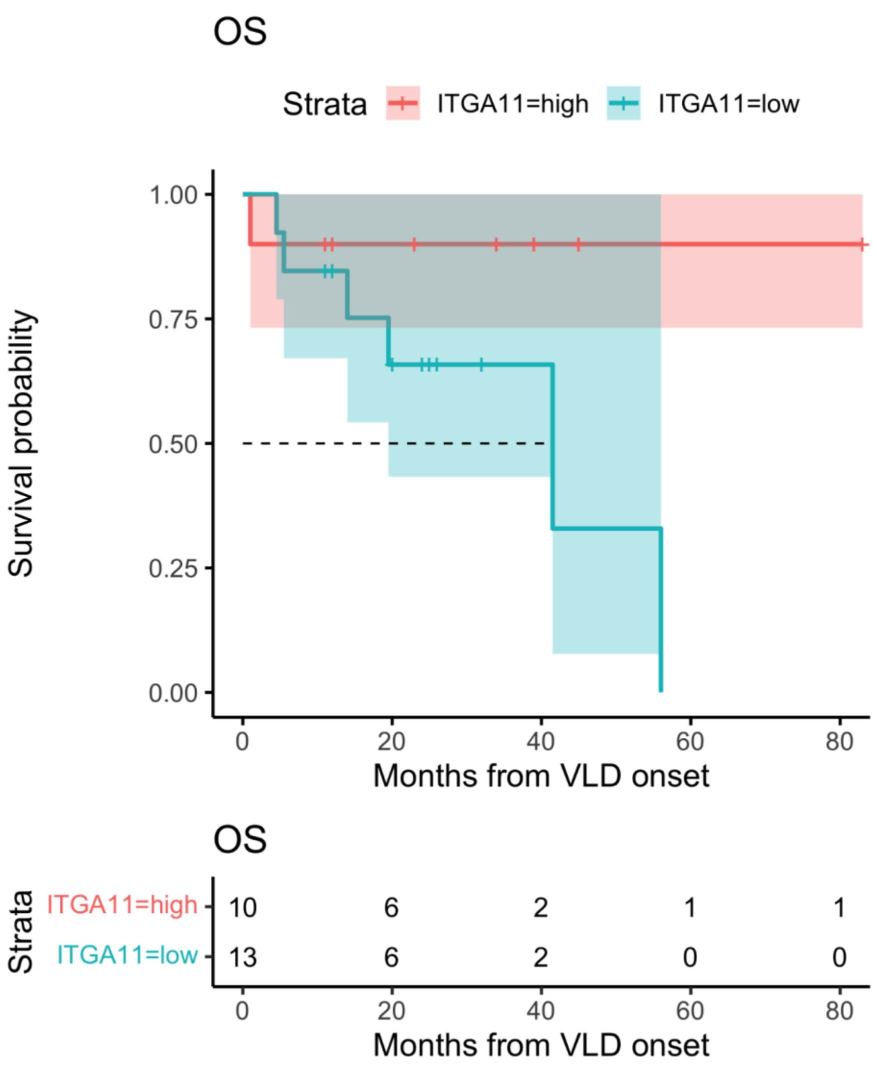

Supplementary Figure S2: Survival analysis dependent on ITGA11-Levels showing a better prognostic overall survival sub-significance (p = 0.147)
